# The levels of circulating tumor DNA and inflammatory proteins depict the clinical response in a patient with metastatic undifferentiated pleomorphic sarcoma, a case report

**DOI:** 10.2340/1651-226X.2025.44337

**Published:** 2025-09-11

**Authors:** Christoffer Vannas, Mandy Escobar, Margaréta Tanyasiová, Mathilda Kindeberg Sederblad, Julia Nyström, Tobias Österlund, David Wennergren, Daniel Andersson, Martin Dalin, Åsa Torinsson Naluai, Henrik Fagman, Anders Ståhlberg

**Affiliations:** a Sahlgrenska Center for Cancer Research, Department of Laboratory Medicine, Institute of Biomedicine, Sahlgrenska Academy at University of Gothenburg, Gothenburg, Sweden; b Department of Oncology, Sahlgrenska University Hospital, Region Västra Götaland, Gothenburg, Sweden; c Department of Clinical Genetics and Genomics, Sahlgrenska University Hospital, Region Västra Götaland, Gothenburg, Sweden; d Wallenberg Centre for Molecular and Translational Medicine, University of Gothenburg, Gothenburg, Sweden; e Department of Orthopaedics, Institute of Clinical Sciences, Sahlgrenska Academy, University of Gothenburg, Gothenburg, Sweden; f Children’s Cancer Centre, Queen Silvia Children’s Hospital, Sahlgrenska University Hospital, Gothenburg, Sweden; g Sahlgrenska Center for Cancer Research, Department of Pediatrics, Institute of Clinical Sciences, Sahlgrenska Academy at University of Gothenburg, Gothenburg, Sweden; h Department of Laboratory Medicine, Institute of Biomedicine, Sahlgrenska Academy at the University of Gothenburg, Gothenburg, Sweden; i Core Facilities, Sahlgrenska Academy at the University of Gothenburg, Gothenburg, Sweden; j Department of Clinical Pathology, Sahlgrenska University Hospital, Region Västra Götaland, Gothenburg, Sweden; k Science for Life Laboratory, Institute of Biomedicine, University of Gothenburg, Sweden

**Keywords:** circulating tumor DNA, liquid biopsy, sarcoma, malignant fibrous histiocytoma, multiomics, proteomics, case report

## Introduction

Undifferentiated pleomorphic sarcoma (UPS) is one of the most frequent high-grade soft-tissue sarcomas [1]. Diagnosis is based on exclusion of an identifiable line of differentiation and other well-defined soft-tissue sarcoma entities [2]. UPS is generally an aggressive malignancy with high risk of metastasis, most commonly to the lungs, bones, and liver [3, 4]. Localized disease is treated with surgical resection, sometimes followed by radiation therapy and/or chemotherapy [5]. Metastatic disease is treated with chemotherapy. Immunotherapy with PD1 inhibitors has shown clinical benefit in later treatment lines, making UPS one of few sarcoma entities where this treatment option is an alternative [2, 6].

Disease and treatment monitoring relies on clinical examinations and radiological assessments, as no validated blood-based markers have shown clinical utility. Circulating tumor DNA (ctDNA), consisting of tumor-derived DNA fragments shed into the blood, has emerged as a promising pan-cancer biomarker for disease monitoring [7]. However, few studies have been conducted on UPS and other high-grade sarcomas [8, 9]. A major challenge when analyzing UPS is the lack of recurrent mutations, limiting the use of generic ctDNA panels. Tumor-informed approaches, where patient-specific mutations are identified through sequencing and then analyzed in blood plasma with ultrasensitive methods such as SiMSen-Seq, may overcome this limitation [10, 11].

Here, we present a case of metastatic UPS where retrospective tumor-informed ctDNA analysis was performed, alongside plasma protein profiling, to explore their value for disease monitoring.

## Case presentation

**Figure 1:**
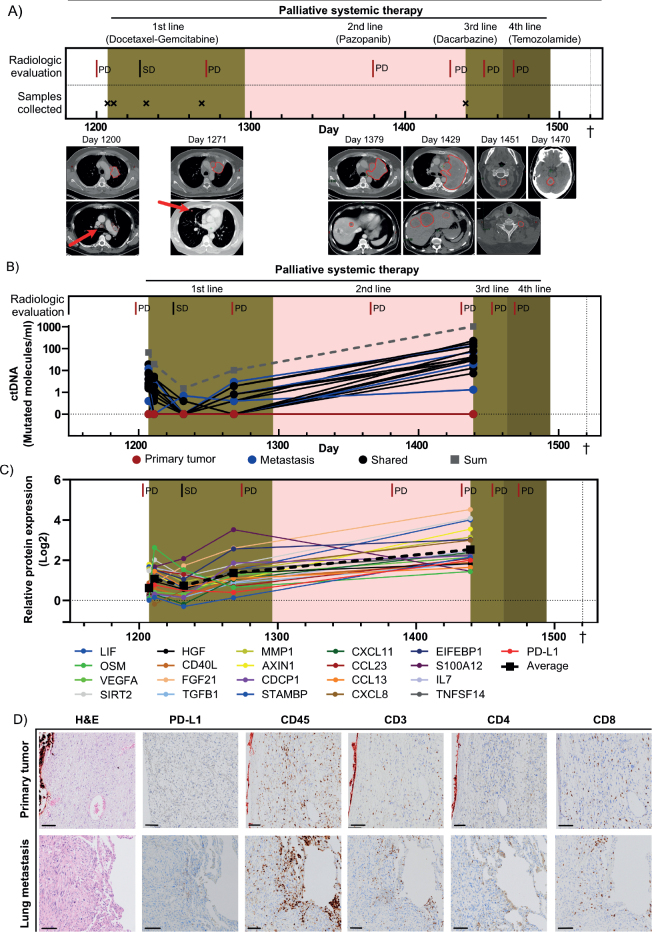
Clinical information, ctDNA data, and blood- and tumor tissue-based inflammatory protein profiling. (A) Timeline showing the clinical course of the patient from initiation of palliative chemotherapy to death. Radiological responses according to RECIST v1.1 and timepoints for blood sampling are shown. Treatments are indicated by colored background. Radiological images at indicated progressive disease are shown below the timeline, with tumors outlined in red. The red arrows indicate tumors that are challenging to detect at this magnification. (B) The level of ctDNA shown as molecules per ml plasma over time for each mutation. The red, blue, and black lines represent the total number of ctDNA molecules for each mutation derived from the primary tumor, the lung metastasis, and mutations shared between the primary tumor and metastasis, respectively. Gray dashed line indicates the sum of all detected ctDNA molecules. (C) Protein expression over time for the 21 inflammatory proteins that were highly expressed at the baseline sample (day 1,207) compared to healthy individuals and upregulated at least two times comparing baseline sample to the last timepoint (day 1,439). Mean expression of all proteins is shown as a dashed black line. (D) Immunohistochemical staining of the primary tumor and the lung metastasis, with antibodies against PD-L1, CD45 (leukocytes), CD3 (lymphocytes), CD8 (cytotoxic lymphocytes), and CD4 (helper T cells). Scale bar is 100 µm. ctDNA: Circulating tumor DNA; PD: Progressive disease; SD: Stable disease. †Death.

A man in his 60s presented in August 2016 with a rapidly enlarging mass in the right proximal thigh. He was otherwise healthy aside from hypertension. Magnetic resonance imaging revealed a 4 × 4.5 cm subcutaneous lesion initially suspected to be a schwannoma (Supplementary Figure 1A, day 0). Surgical excision revealed high-grade UPS (Supplementary Figure 1B). Subsequent resections confirmed residual tumor, and wide resection margins were achieved only after the third resection. The patient then received adjuvant radiotherapy. Two years later, a new lesion was detected in the left lung, which was resected and confirmed as metastatic UPS (Supplementary Figure 1A day 684, Supplementary Figure 1B). Six cycles of adjuvant chemotherapy with doxorubicin–ifosfamide were given. Six months after completing chemotherapy, radiological imaging revealed a new lesion in the left lung hilus (Supplementary Figure 1A day 1,168, Supplementary Figure 1B). Bronchoscopic cytology confirmed malignancy, but surgery was not feasible due to additional findings of lung and lymph node involvement (Figure 1A, day 1,200). Palliative chemotherapy was initiated with docetaxel-gemcitabine, initially with good response. After three cycles, a new lesion appeared in the right lung (Figure 1A, day 1,271), and treatment was switched to pazopanib. Disease progression occurred after 5 months (Figure 1A, day 1,429), after which dacarbazine and subsequently temozolomide were administered, without any response (Figure 1A, day 1,451 and 1,470). The patient ultimately died.

The patient was included in the SARKOMtest study at initiation of palliative chemotherapy, and blood samples were collected during palliative treatment. Whole exome sequencing was performed on both the primary tumor and the first lung metastasis, finding 892 and 219 mutations, respectively, with 66 mutations shared. Twenty-three mutations, five unique to the primary tumor (variant-allele frequencies 21.4–32.7%), five unique to the metastasis (variant-allele frequencies 31.6–40.0%), and 13 shared (variant-allele frequencies 14.3–72.7%), were selected for retrospective tumor-informed ctDNA analysis in blood plasma using SiMSen-Seq (Supplementary Table 1). More than 80% of the mutations unique to the metastasis and shared mutations were detectable in plasma, whereas no mutations unique to the primary tumor could be detected (Figure 1B, Supplementary Figure 2A–C). Overall, 61% of the mutations present in the primary tumor, including those shared with the metastasis, were detectable in ctDNA. The total levels of ctDNA demonstrated a consistent trend with radiological outcomes and total tumor volume (Supplementary Figure 2D). Mutations with high allele frequencies in tumor tissue generally showed higher ctDNA levels (Supplementary Figure 3A–D).

We then analyzed the expression levels of 92 inflammatory proteins in the same blood samples, using proximity extension assay. The mean expression of the inflammatory proteins increased as the disease progressed (Supplementary Figure 3E). Twenty-one proteins were selected for further analysis, based on elevated levels compared to healthy controls at baseline (day 1,207) as well as > 2-fold upregulation from baseline to day 1,439. These proteins included mediators of T-cell activation (AXIN1, CD40, TNFSF14, IL7, and CXCL11) as well as T-cell suppression (PD-L1 and TGFB1), and their mean levels correlated with clinical response (Figure 1C, Supplementary Table 1). A reassessment of the primary tumor and the lung metastasis confirmed that they were PD-L1 negative, though tumor-infiltrating lymphocytes (CD3+ and CD45+) were present, with more CD8+ cytotoxic T cells than CD4+ helper T cells (Figure 1D).

## Discussion

This case illustrates the utility of ctDNA as a minimal invasive biomarker for monitoring UPS. The levels of ctDNA correlated well to radiological response, offering potential as an indicator for treatment response. In contrast to previous studies [10, 11], we observed no lead time between increased ctDNA levels and radiological progression, possibly due to long time between blood samplings. Importantly, this case showed that tumor-informed ctDNA panels could be generated using sequencing data from either a primary tumor or a metastatic site. Mutations unique to the metastasis and shared mutations displayed high probability (> 80%) of detection, whereas mutations unique to the primary tumor could not be detected, possibly due to clonal evolution and intratumoral heterogeneity. Furthermore, mutations with high variant-allele frequencies in tumor tissues corresponded to higher ctDNA levels in plasma.

Evaluation of inflammatory protein profiling added complementary insights. Disease progression was associated with mediators of both T-cell activation and suppression. In particular, the observed increase in soluble PD-L1 was interesting, given the established utility of PD-1 checkpoint inhibitors in UPS [6, 12]. While tissue expression of PD-L1 is generally associated with improved responses to PD-1 checkpoint inhibitors [13, 14], elevated levels of soluble PD-L1 have been linked to poorer outcomes and reduced treatment efficacy [15–17]. The absence of PD-L1 in the patient’s tumor tissue in combination with high levels of soluble PD-L1 indicates a low probability of response to immune checkpoint inhibition. However, this remains hypothetical, as the patient never received this treatment.

## Conclusions

This case demonstrated the utility of ctDNA quantification using tumor-informed panels to monitor the clinical response in a patient with UPS. Panels should, if possible, contain mutations shared between the primary tumor and metastatic lesions for optimal sensitivity. If sequencing data are limited to the primary tumor, the panel should target multiple mutations with high variant-allele frequencies. Simultaneous quantification of plasma proteins could provide additional insights into the tumor biology. Further studies with more patients and samples are needed to determine clinical utility of blood-based biomarker analysis in UPS.

## Material and methods

All material and methods can be found in the supplementary file ‘Material and methods’.

## Supplementary Material







## Data Availability

The sequencing data generated during the current study are not publicly available due to the restrictions imposed by the EU General Data Protection Regulation (GDPR) and applicable personal data protection laws. De-identified summary data (e.g. mutation lists, treatment timelines, ctDNA trends, and proteomic signature results) are included within the article and its supplementary materials. Further de-identified or aggregate data may be made available by the corresponding author upon reasonable request and subject to formal data-sharing agreements, ethics approval, and institutional data protection oversight to ensure compliance with GDPR and related regulations.
