# Supplementary material for: The levels of circulating tumor DNA and inflammatory proteins depict the clinical response in a patient with metastatic undifferentiated pleomorphic sarcoma, a case report
Source: Acta Oncol. 2025 Sep 11;64:44337. doi: 10.2340/1651-226X.2025.44337 (PMC12439215; doi:10.2340/1651-226X.2025.44337)
Supplement: Supplementary file 1 [file AO-64-44337-s1.pdf]

Supplementary material has been published as submitted. It has not been copyedited, or typeset by Acta Oncologica

Blood sample collection

The patient participated in the study SARKOMtest and had signed an informed consent. The study was approved by the Regional Ethical Review Board in Gothenburg Dnr 485-16 with amendments T-795-15, T525-18 and 2021-04895. Blood plasma from healthy controls were obtained from the PREAN study, approved by the Regional Ethical Review board in Gothenburg Dnr 054-15. Blood samples were collected in EDTA tubes and plasma was isolated within two hours using centrifugation at 2000g for 10 minutes. Plasma and buffy coat were stored at -80°C until further use.

Whole exome sequencing

Tumor DNA was extracted from formalin-fixed paraffin-embedded material using GeneRead FFPE DNA kit, and DNA from blood cells was extracted using QIAamp DNA Blood Mini Kit (both Qiagen) according to manufacturer's instructions. Whole exome sequencing was performed by the SNP&SEQ Technology Platform, Uppsala, Sweden. Raw fastq-files were processed by first trimming off sequencing adapters using cutadapt [1] with a Phred score cutoff of 20. The reads were used as input to the Sarek pipeline [2] in somatic variant calling mode with the tumor sample matched to the DNA sample prepared from blood cells as previously described [3]. Based on whole exome sequencing data, we designed a SiMSen-Seq panel targeting 23 mutations with high variant allele frequencies.

Gene-set enrichment analyses of the 500 genes with mutations with the highest variant allele frequencies in the primary tumor and the 219 genes with detected mutations in the first lung metastasis respectively, were performed using GenePattern 2.0 [4].

SiMSen-Seq

Circulating cell-free DNA was extracted from blood plasma using the QIAsymphony SP system with the QIAsymphony DSP Circulating DNA Kit (Qiagen), according to the manufacturer's instruction. A second centrifugation was performed at 2000 g for 10 minutes at room temperature prior to cfDNA

extraction. Concentration of DNA was quantified with Qubit 3.0 Fluorometer (Thermo Fisher Scientific). SiMSen-Seq was used to analyze DNA as previously described [5] with minor adjustments. The experimental protocol consisted of two PCR steps. In the first step, target molecules were barcoded with unique molecular identifiers. In the second step, target products were amplified with indexed Illumina adapters. Library quality was assessed with a 5200 Fragment Analyzer System with the HS NGS Fragment Kit (both Agilent technologies). Libraries were pooled and purified using the Pippin Prep DNA Size Selection System with the 2% Agarose kit (Sage Science). Sequencing was performed on the MiniSeq Sequencing System using 20% PhiX control v3 and High Output Reagent Kit (all Illumina) in single-end 150 bp mode with a final library concentration of 1.3 pM. The SiMSen-Seq sequencing data were analyzed using the UMIErrorCorrect pipeline v0.29 [6] with parameters –umi-length 10, --spacer-length 16 and -Q 10. The reads were aligned to the hg38 reference genome and a bed file with genomic coordinates of the patient-specific assays were used to constrain variant calling and for annotation.

Proximity Extension Assay

Approximately 20 µL of each blood sample was analyzed using proximity extension assay with the Inflammatory Target 96 panel (both Olink) at TATAA Biocenter, Gothenburg, Sweden. Normalized protein expression was used for all analyses where the expression level of each protein was normalized to the mean expression of ten blood plasma samples from healthy controls. All figures were created using GraphPad Prism 10.3.2 (GraphPad).

Tumor volume analysis

Radiological response to treatment was retrospectively evaluated according to RECIST 1.1. Volumetric assessments were performed using the ARIA research module (Varian Medical System). In brief, diagnostic radiological images were imported into the software, the outlines of each detected tumor in axial sections were contoured and corresponding 3D-structures were interpolated and volumes calculated. All figures were created using GraphPad Prism 10.3.2.

References

1. Martin M., *Cutadapt removes adapter sequences from high-throughput sequencing reads*. 2011, 2011. **17**(1): p. 3 %J EMBnet.journal.
2. Garcia M., Juhos S., Larsson M., Olason P.I., Martin M., Eisfeldt J., et al., *Sarek: A portable workflow for whole-genome sequencing analysis of germline and somatic variants*. F1000Res, 2020. **9**: p. 63.
3. Micallef P., Santamaría M.L., Escobar M., Andersson D., Österlund T., Mouhanna P., et al., *Digital sequencing is improved by using structured unique molecular identifiers*. Genome Biol, 2025. **26**(1): p. 37.
4. Reich M., Liefeld T., Gould J., Lerner J., Tamayo P. and Mesirov J.P., *GenePattern 2.0*. Nature Genetics, 2006. **38**(5): p. 500-501.
5. Ståhlberg A., Krzyzanowski P.M., Egyud M., Filges S., Stein L. and Godfrey T.E., *Simple multiplexed PCR-based barcoding of DNA for ultrasensitive mutation detection by next-generation sequencing*. Nat Protoc, 2017. **12**(4): p. 664-682.
6. Österlund T., Filges S., Johansson G. and Ståhlberg A., *UMIErrorCorrect and UMIAnalyzer: Software for Consensus Read Generation, Error Correction, and Visualization Using Unique Molecular Identifiers*. Clin Chem, 2022. **68**(11): p. 1425-1435.
