# Supplementary material for: The levels of circulating tumor DNA and inflammatory proteins depict the clinical response in a patient with metastatic undifferentiated pleomorphic sarcoma, a case report
Source: Acta Oncol. 2025 Sep 11;64:44337. doi: 10.2340/1651-226X.2025.44337 (PMC12439215; doi:10.2340/1651-226X.2025.44337)

Supplementary material has been published as submitted. It has not been copyedited, or typeset by Acta Oncologica

Supplementary Figure 1: **Clinical information and radiological data.** A) Timeline showing the clinical course from the first surgery of the primary tumor to death. Top, radiological responses according to RECIST v1.1; middle, blood sampling for ctDNA/proximity extension assay analysis; bottom, tumor tissue used for whole exome sequencing. †, Death. Radiological images at diagnosis and recurrences are shown above the timeline. Red arrows denote tumors. B) Histological sections of the primary tumor in the thigh (left) and the lung metastasis (right). Red scale bar is 2000 µm, black scale bar is 100 µm. The right images are enlargements of the areas indicated by the frame in the left images.

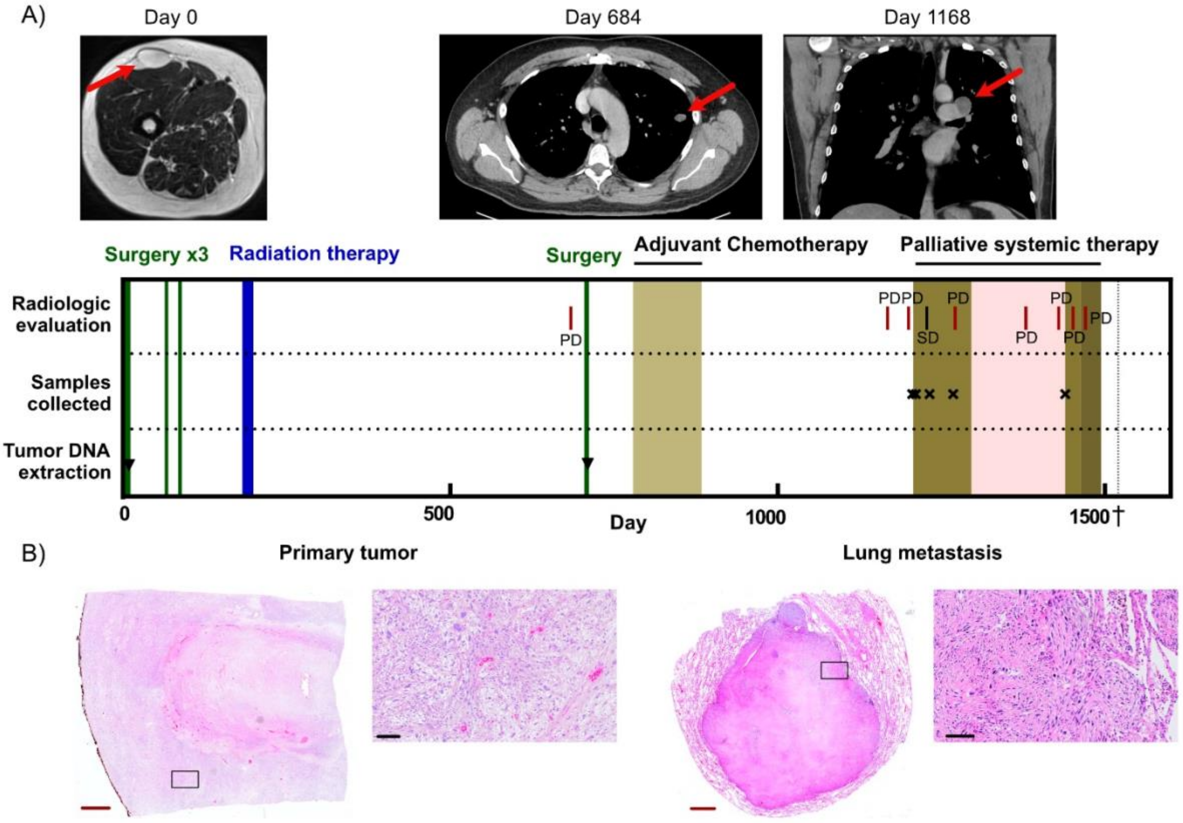

Supplementary Figure 2: **Additional ctDNA data.** A-C) The levels of ctDNA shown as molecules per ml plasma over time, for mutations expressed exclusively in the primary tumor (A, n=5), lung metastasis (B, n=5) or mutations shared by the primary tumor and metastasis (C, n=13). The box indicates the number and percentage of mutations being detectable at any timepoint for each sub-ctDNA panel. D) The total level of ctDNA shown as molecules per ml plasma and radiological total tumor volume. Gray line represents the total number of detected ctDNA molecules for all mutations. The black dashed line indicates radiological total tumor volume, reported as cm^3. †, Death.

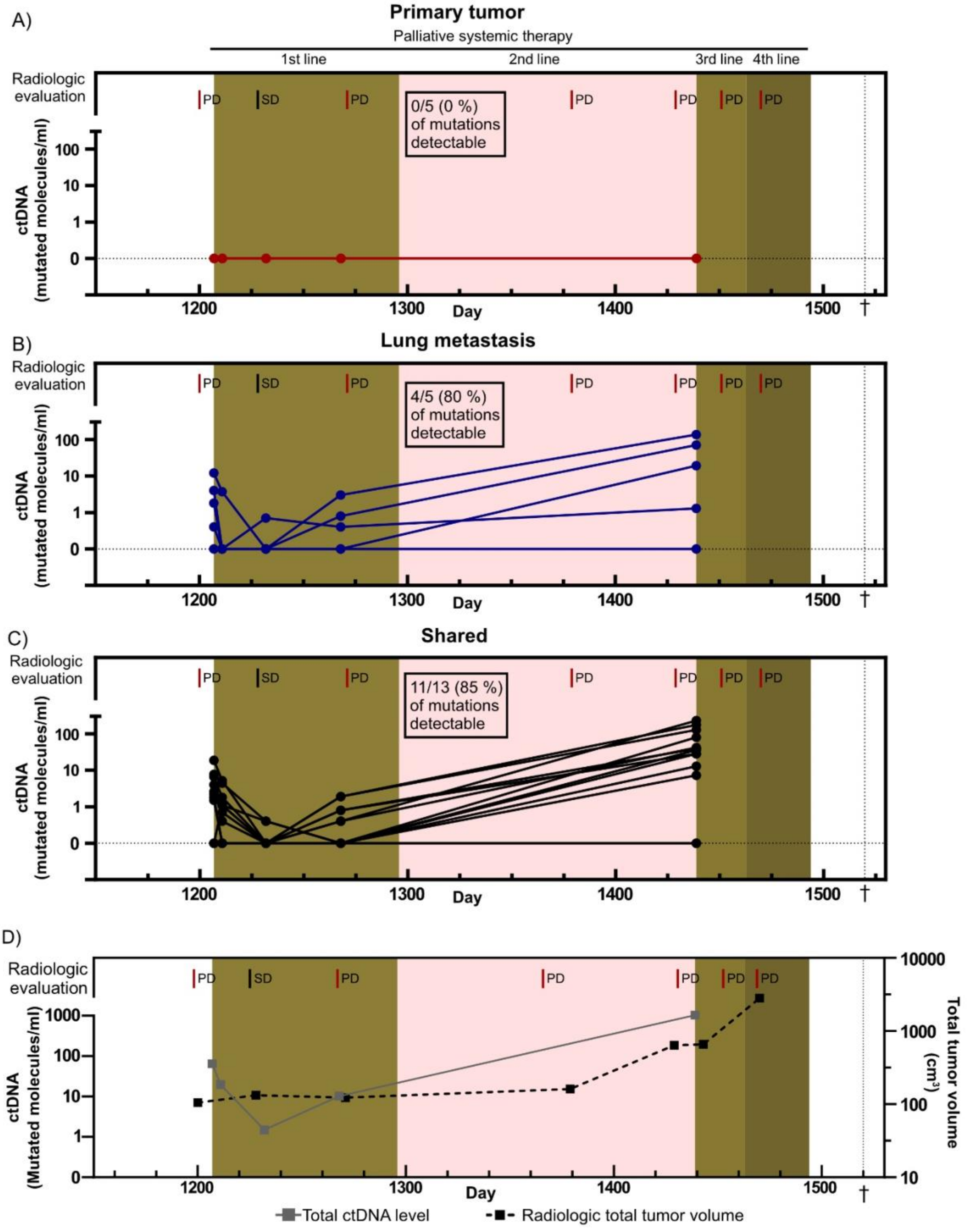

Supplementary Figure 3: **Variant allele frequencies in tumor and plasma. Additional protein data.** Pearson correlation analyses comparing the variant allele frequencies (VAF) in the primary tumor and in blood at the first (P1) timepoint (A) and the last (P5) timepoint (B) and the VAF in the lung metastasis and in blood at timepoint P1 (C) and P5 (D). The red, blue and black dots indicate mutations derived from the primary tumor, the lung metastasis, and mutations shared between the primary tumor and the metastasis, respectively. Pearson correlation coefficient (r) was calculated where p<0.05 was considered statistically significant. D) Relative expression of 92 inflammation-related proteins. Data are normalized to the expression in five healthy individuals. Red dots indicate proteins selected for in-depth analysis in Figure 1C.

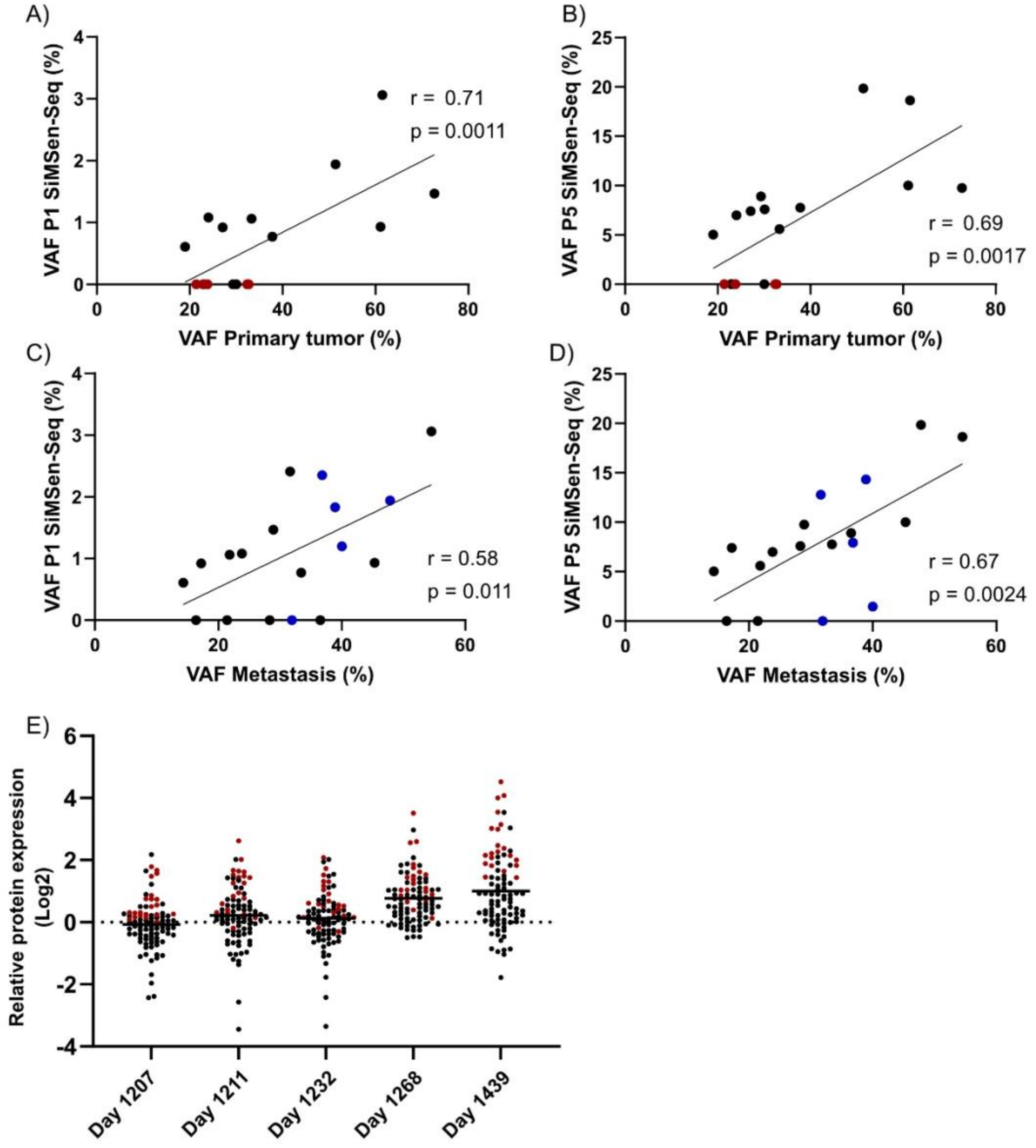

Supplement: Supplementary file 2 [file AO-64-44337-s2.pdf]
